# Streamlining ADHD Annual Reviews: Implementation of a Form-Based System to Replace Face-to-Face Consultations

**DOI:** 10.1192/bjo.2025.10360

**Published:** 2025-06-20

**Authors:** Mike Smith, Debbie-Faith Ebeye, Sumir Punnoose, Rudi Nilsen, Emma Jackson

**Affiliations:** Leeds and York Partnership NHS Foundation Trust, Leeds, United Kingdom

## Abstract

**Aims:** Patients prescribed medication for ADHD require an annual review, generally conducted by specialist services, which accounts for a significant proportion of the workload. Delays in annual reviews can lead to GPs withdrawing Shared Care and discontinuing medication. Optimising this process could release much-needed resources for already struggling ADHD services.

This project evaluated the impact of replacing routine face-to-face annual reviews (ARs) with a streamlined, form-based system, with key objectives of assessing improvements in service efficiency, patient outcomes, and resource allocation while maintaining adherence to NICE guidelines.

**Methods:** A single-page Adult ADHD-friendly form consistent with NICE Guidelines on annual reviews was developed to assess medication adherence, symptom stability, and the appropriateness of continued ADHD medication. Created with a service user panel, the form was designed to allow patients to complete it by phone or email in less than 3 minutes.

Following a review of the responses on the AR form, patients requiring a further review or intervention were offered clinic appointments. Data from January to June 2023 were analysed to determine the proportion of patients requiring follow-up, and care records for this group were reviewed.

**Results:** Of 288 patients contacted, 262 responded, with only 60 (20%) requiring a follow-up review, mainly for medication effectiveness issues (37.1%), dose adjustments (22.6%), or side effects (17.7%), indicating that 80% of cases were manageable via the form alone.

Only 2 forms were redone due to incompletion. 25 patients (8.7%) did not respond, and were discharged after further attempts, including GP contact.

Extrapolated data: Approximately 700 patients were on the AR list. Replacing routine 1-hour face-to-face reviews with 5-minute paper reviews for 80% of patients saved an estimated 560 patient hours annually. This enabled an additional 112 assessments for new or complex cases (assuming each assessment takes 5 hours).

Consultant workload analysis:

Each Programmed Activity (PA) equates to 4 hours. 560 hours = 140 PAs saved annually, or 23 weeks of full-time consultant time (based on 6 clinical PAs per week). At an average consultant salary of £118,000/year, this system achieved a cost saving of approximately £60,000 annually.

**Conclusion:** This innovative approach demonstrates that replacing routine face-to-face ADHD reviews with a form-based system significantly enhances service efficiency, reduces waiting times, and optimises resources. Positive feedback from patients suggests high acceptability, with many valuing the convenience of avoiding unnecessary clinic visits. This system aligns with NICE guidelines by ensuring timely reviews while preventing service bottlenecks.

